# Comprehensive Comparative Analysis of the *JAZ* Gene Family in Common Wheat (*Triticum aestivum*) and Its D-Subgenome Donor *Aegilops tauschii*

**DOI:** 10.3390/plants13091259

**Published:** 2024-04-30

**Authors:** Zhiwen Zhai, Yuqing Che, Shuaifeng Geng, Shaoshuai Liu, Shuqin Zhang, Dada Cui, Zhongyin Deng, Mingxue Fu, Yang Li, Xinyu Zou, Jun Liu, Aili Li, Long Mao

**Affiliations:** 1 National Key Facility for Crop Resources and Genetic Improvement, Institute of Crop Science, Chinese Academy of Agricultural Sciences, Beijing 100081, China; arvin16@126.com (Z.Z.); 15670535279@163.com (Y.C.); gengshuaifeng@caas.cn (S.G.); liushaoshuai@caas.cn (S.L.); cuidada2018@163.com (D.C.); 15093595930@163.com (Z.D.); fumingxue0125@126.com (M.F.); z1143445173@163.com (Y.L.); 82101205039@caas.cn (X.Z.); liujun@caas.cn (J.L.); 2 State Key Laboratory of Plant Physiology and Biochemistry, Department of Plant Genetics and Breeding, National Center for Evaluation of Agricultural Wild Plants (Rice), China Agricultural University, Beijing 100094, China; zhangshq22@cau.edu.cn

**Keywords:** *Aegilops tauschii*, wheat, *JAZ* gene family, tandem duplication, yield

## Abstract

JASMONATE-ZIM DOMAIN (JAZ) repressor proteins work as co-receptors in the jasmonic acid (JA) signalling pathway and are essential for plant development and environmental adaptation. Despite wheat being one of the main staple food crops, until recently, comprehensive analysis of its *JAZ* gene family has been limited due to the lack of complete and high-quality reference genomes. Here, using the latest reference genome, we identified 17 *JAZ* genes in the wheat D-genome donor *Aegilops tauschii*. Then, 54 *TaJAZs* were identified in common wheat. A systematic examination of the gene structures, conserved protein domains, and phylogenetic relationships of this gene family was performed. Five new *JAZ* genes were identified as being derived from tandem duplication after wheat divergence from other species. We integrated RNA-seq data and yield QTL information and found that tandemly duplicated *TaJAZ* genes were prone to association with spike-related traits. Moreover, 12 *TaJAZ* genes were located within breeding selection sweeps, including 9 tandemly duplicated ones. Haplotype variation analysis of selected *JAZ* genes showed significant association of *TaJAZ7A* and *TaJAZ13A* with thousand-grain weight. Our work provides a clearer picture of wheat *JAZ* gene evolution and puts forward the possibility of using these genes for wheat yield improvement.

## 1. Introduction

Bread wheat is one of the main staple crops whose production is nowadays being challenged by the changing climate. Use of wild germplasms, such as *Aegilops tauschii* (2n = 14, DD), a D-genome donor of hexaploid common wheat (*Triticum aestivum* L.; 2n = 6x = 42, AABBDD), is one the most efficient approaches to produce more resilient wheat varieties. *Ae. tauschii* carries valuable genetic resources related to tolerance to biotic and abiotic stresses that are essential for crop breeding [1]. The prerequisite for *Ae. tauschii* to be used for common wheat improvement is its highly similar genome to the D subgenome of bread wheat [2,3]. In addition to its role in genetics and genomics, *Ae. tauschii* is also considered a model plant to explore the evolution and diversification of *Triticeae* plants [4].

The lipid-derived hormone jasmonic acid (JA) is an essential phytohormone that regulates plant growth, development, and defense [5,6]. The bioactive jasmonoyl-isoleucine (JA-Ile) is perceived by a receptor complex containing the proteins CORONATINE INSENSITIVE (COI1) and JASMONATE ZIM-domain (JAZ) [7,8]. The JA signal promotes the specific binding of COI1 and JAZ proteins, leading to ubiquitination of JAZ via SCF^COI1^ and subsequent degradation through the 26S proteasome [7,9].

The JAZ protein family belongs to the TIFY transcription factor superfamily and is characterized by two functional domains, TIFY (also known as ZIM) and Jas (also known as CCT_2) [5]. The TIFY domain, typically consisting of 28 amino acids located at the N-terminus of JAZ protein sequences, contains a core sequence of TIF[F/Y]XG [10]. In the JA signaling pathway, this domain facilitates interaction between the JAZ protein and its co-suppressor, NINJA, which together restrict JA signal transduction. The Jas domain, located near the C-terminus of the JAZ protein sequences, is highly conserved among family members, with 10 amino acids being identical or replaced in conservation. Its role is to mediate direct binding between the JAZ protein and MYC2 in the JA signaling pathway, inhibiting *MYC2* transcriptional activity and further restricting the expression of JA response genes. 

The model plant *Arabidopsis* has 12 *JAZ* genes [11]. The crops rice, maize, and sorghum have 15 [12], 16 [13], and 21 *JAZ* genes [14], respectively. It has been shown that each *JAZ* gene within the family has a distinct biological function. For instance, *OsJAZ9*-mediated JA signaling enhances tolerance to salt in rice [15], while overexpressing modified *OsJAZs* can result in flower organ malformation in rice [16]. In cotton, GhJAZ1 activated *GbWRKY1* and led to increased resistance against *Verticillium dahlia* [17]. 

Although the *JAZ* gene family has been studied at various levels [18,19,20], there have been limitations due to the incomplete hexaploid wheat reference genome, which was not made available until 2018 [21]. Using the latest annotated reference genome, this study conducted a comprehensive characterization of the *JAZ* gene family in wheat and its DD subgenome donor *Ae. tauschii.* We then took advantage of published transcriptome data as well as the data generated in this work to study the expression patterns of wheat genes during yield-related trait development, particularly the structurally featured tandemly duplicated *JAZ* genes. Haplotype analysis revealed a close association between *JAZ* genes and wheat yield traits. Thus, our work demonstrated the potential application of *JAZ* genes in wheat molecular breeding.

## 2. Results

### 2.1. Identification of JAZ Genes in Common Wheat and Its D-Genome Donor Ae. tauschii

**Figure 1:**
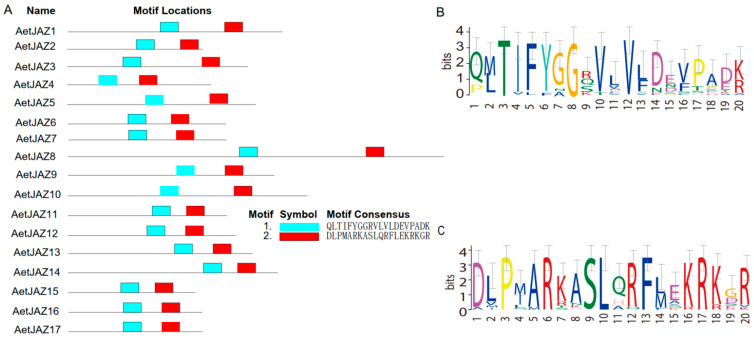
Characterization of JAZ genes in *Ae. tauschii*. (**A**) Domain distribution among AeJAZ proteins. (**B**) Domain distribution among TaJAZ proteins. (**C**) The consensus sequence of the TIFY domain from AeJAZ proteins..

With the most recently updated reference genomes, a total of 17 *JAZ* genes were identified in *Ae. tauschii* (*AeJAZ*). As expected, wheat JAZ proteins contained two conserved domains, TIFY and Jas (Figure 1A). The core motif for the TIFY domain was “TI[F/V]YXG” (Figure 1B), while the Jas domain had a signature sequence of “SLX2FX2KRX2RX7PY” (Figure 1C). Both domains are characteristics of JAZ proteins and are highly conserved. The residues in these domains are in fact conserved among all species studied, as shown by the sequence logo. Despite this, some proteins appear to lack the PY motif at the end of the Jas domain, although the motif may not be needed for ligand-dependent COI1-JAZ interaction [10], while the TIFY domain is highly conserved.

As shown in Table S1, *AeJAZ* genes ranged from 449 to 2299 bp at the genomic level, while their proteins varied from 142 to 419 amino acids. Genomic structure analysis revealed that the number of introns in these genes ranged from zero to six, with nearly half of the genes having no intron. Except for *AetJAZ4*, intronless genes were closely positioned on the same chromosome, possibly due to tandem duplication. Subcellular localization results indicated that the majority of gene proteins were located in the nucleus on the subcellular level. However, only one JAZ protein was predicted to be located in chloroplasts.

### 2.2. Expansion of the JAZ Gene Family in Monocots

**Figure 2:**
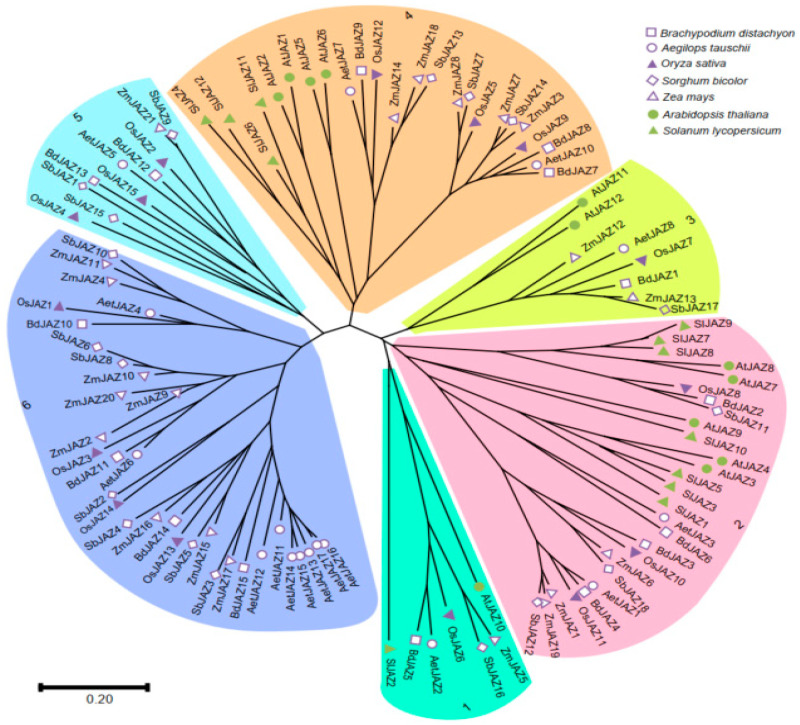
Phylogenetic tree of studied plant *JAZ* gene proteins. Different colors represent different groups. *Aet*: *Ae. tauschii*, *Bd*: *B. distachyon*, *Os*: *O. sativa*, *Sb*: *S. bicolor*, *Zm*: *Z. mays*, *At*: *A. thaliana*, *Sl*: *S. lycopersicum*.

As shown previously, dicotyledonous plants, such as *Arabidopsis* and tomato, contain 12 *JAZ* genes [11]. The number is significantly increased in monocotyledonous plants. For example, the rice genome has 15 *JAZ* genes, while other monocot plants like *Brachypodium*, *Ae. tauschii*, sorghum, and maize have 15, 17, 18, and 21 *JAZ* genes, respectively. Phylogenetic analysis showed that *JAZ* genes can be divided into six branches (Figure 2).

**Figure 3:**
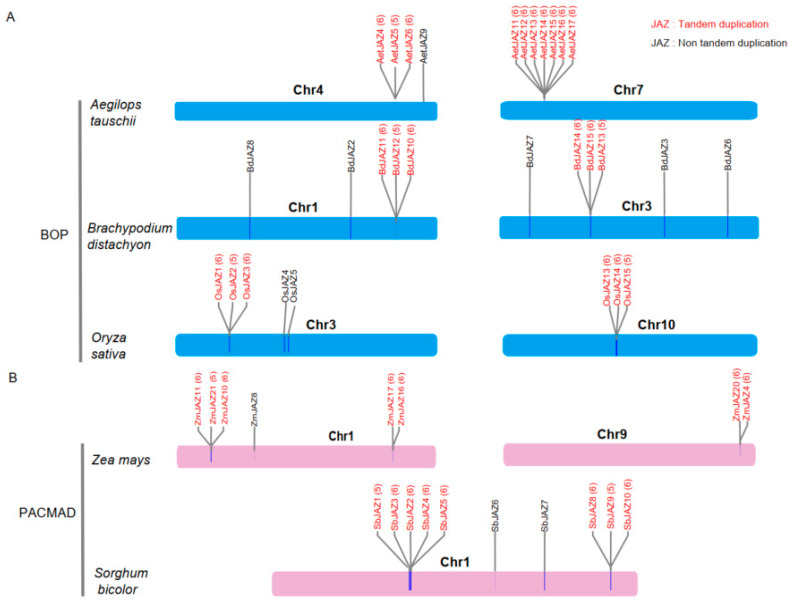
Different amplification patterns in *JAZ* genes between PACMAD (Panicoideae, Aristidoideae, Chloridoideae, Micrairoideae, Arundinoideae, and Danthonioideae) and BOP (Bambusoideae, Oryzoideae, and Pooideae) species. (**A**) Close relationships among *AeJAZ* genes suggest tandem duplication. (**B**) *JAZ* genes in PACAMD arose by both tandem duplication and dispersed duplication.

Notably, branches 5 and 6 were specific to monocotyledonous plants. *JAZ* genes in dicotyledonous plants were only found in branches 1, 2, 3, and 4, where the relationships between *JAZ* genes within each branch aligned with the evolutionary relationships of the species. The monocot unique clade indicated monocot-specific *JAZ* gene expanding events. Unlike maize *JAZ* genes with a higher number of proximal duplicated pairs and dispersed duplicated pairs, *AeJAZ* genes were more frequently amplified through tandem repeats (Figure 3). Interestingly, tandemly duplicated genes on chromosome 7 were closely clustered in the phylogenetic tree of *Ae. tauschii*, whereas tandemly repeated genes on chromosome 4 were dispersed and more similar to their orthologous genes from other species, indicating that these duplication events occurred before the divergence of these species.

### 2.3. Functional Differentiation of JAZ Genes under JA Induction

**Figure 4:**
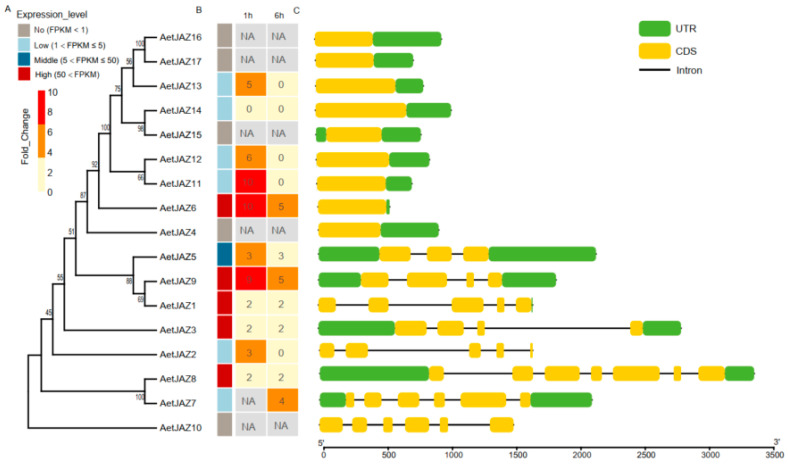
Response of *AeJAZ* genes to JA treatment. (**A**) Phylogenetic tree of *AeJAZ* genes. (**B**) Expression patterns of *AeJAZ* genes under JA treatment. (**C**) Gene structures of *AeJAZ* genes.

To study the possible biological functions of the expanded JAZ genes in monocots, we used *AeJAZ* genes as examples. RNA-seq data collected at 1 h and 6 h after treatment with JA showed 1252 differentially expressed genes (DEGs) (Figure S1), indicating that genes were sequentially induced by JA. At 1 h, 324 DEGs were identified relative to mock treatment, whereas at 6 h, 1252 DEGs were identified (Figure S2C). We found that four *AeJAZ* genes (*AeJAZ*3, *AeJAZ*6, *AeJAZ*8, and *AeJAZ*10) were highly expressed genes over the course of 1 h to 6 h of JA treatment, while lowly expressed *AeJAZ* genes (*AeJAZ*13, *AeJAZ*12, *AeJAZ*11, *AeJAZ*2, and *AeJAZ*7) could be detected either at 1 h or 6 h, but not both time points. Five *AeJAZ* genes (*AeJAZ*15, *AeJAZ*16, *AeJAZ*17, *AeJAZ*4, and *AeJAZ*10) were not detected for expression, with the first four of them (*AeJAZ*15, *AeJAZ*16, *AeJAZ*17, and *AeJAZ*4) being tandemly repeated genes with no introns, while *AeJAZ*10 had multiple introns (Figure 4). Such an observation indicated that newly arisen genes tend to be not expressed, or at least not in the time duration studied, while better-evolved ones may not always be expressed.

### 2.4. JAZ Genes Displayed Imbalanced Subgenome Duplication in Wheat

**Figure 5:**
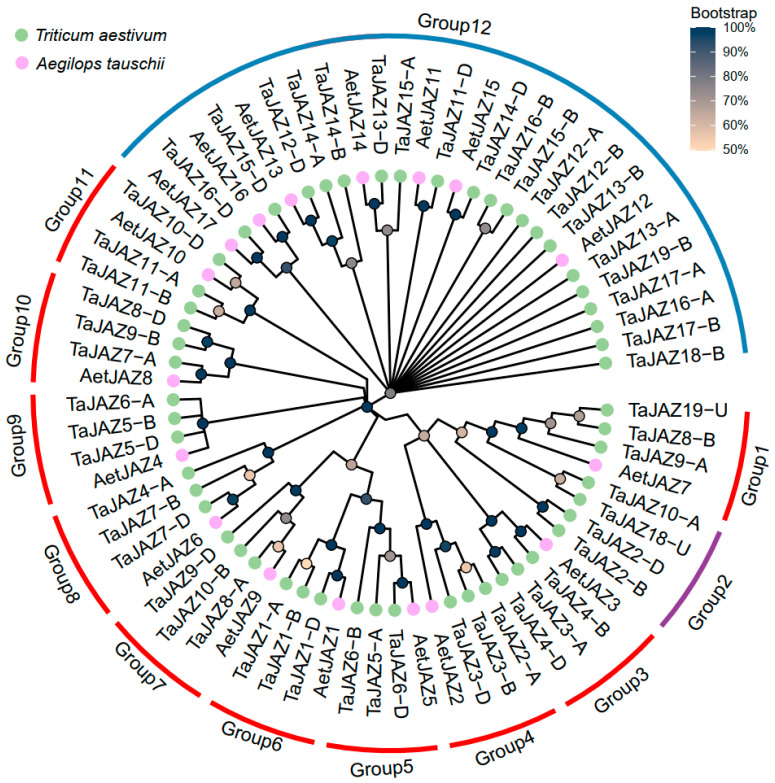
Phylogenetic analysis of *AeJAZ* and *TaJAZ* genes. Red arc indicates orthologous group of three TaJAZ homoeologs and one *AeJAZ* gene. Blue arc indicates groups with no distinct patterns. The purple arc indicates groups of wheat *JAZ* genes only.

Ae. tauschii serves as the DD subgenome donor for hexaploid wheat. To study the contribution of *AeJAZ* genes to the wheat genome and probably wheat breeding, we compared JAZ genes between diploid Ae. tauschii and hexaploid wheat. We found a total of 54 JAZ genes in hexaploid wheat, with 17 of them from the A subgenome, 19 from the B subgenome, and 16 from the D subgenome (Figure S1). There were two genes (TaJAZ18-U and TaJAZ19-U) that were located on unidentified scaffolds of the reference genome (CS V2.0). Relative to the 17 *AeJAZ* genes, most of the wheat JAZ genes were located at corresponding orthologous regions (Figure 5). Phylogenetic tree analysis also supported their correspondence with wheat JAZ genes that were clearly clustered with *AeJAZ* genes, and together they formed eleven orthologous groups. Interestingly, some genes within the same subgenome were closely clustered together rather than being closely adjacent to the other two subgenomes, indicating recent gene expansion within the subgenome. 

Judging from the phylogenetic relationships, we found that in the ancestral DD genome, the expansion events of *JAZ* genes occurred within the DD subgenome. In other words, these genes may have gone through expansion prior to the formation of hexaploid wheat. On the other hand, we observed *JAZ* genes in group 2 (*TaJAZ2-B*, *TaJAZ2-D*, *TaJAZ10-A*, and *TaJAZ18-U*) with no corresponding orthologs, which was probably caused by gene loss events if it was not an artifact of misassembly of the reference genome (Figure 5). These results indicated that some genes underwent amplification in the ancestral diploid, while others kept expanding in the evolution and domestication process of hexaploid wheat. Further investigation is needed to explore the roles of these expanded and conserved *TaJAZ* genes in the context of wheat growth and development.

### 2.5. JAZ Genes within QTL Intervals May Be Associated with Spike Development

To explore possible functions of *TaJAZ* genes in wheat, we checked their genomic loci regarding the QTL intervals identified. As shown in Supplementary Table S1, we identified 28 *TaJAZ* genes located within 14 QTLs associated with spike development, including 10 QTLs for the number of spikelets per spike (NSS), 3 QTLs influencing grain number per spike (GPS), and 1 QTL for flowering time or heading date (HD). Among triplet genes (those with homoeologs present in all three subgenomes), some of them had one homeolog located within the QTL interval, while the remaining two were not. 

**Figure 6:**
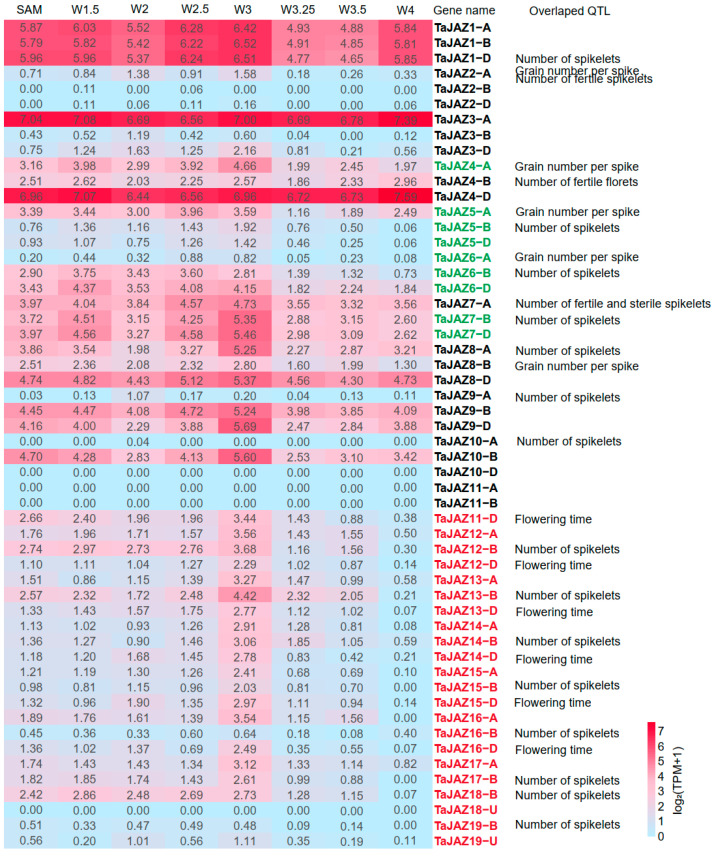
Expression patterns of *TaJAZ* genes at various stages of wheat spike development. Genes in green are derived from tandem duplication on chromosome 4. Genes in red are tandemly duplicated ones on chromosome 7.

We then examined the expression patterns of all *TaJAZ* genes in the spikes for possible functional differentiation or pseudogenization using public transcriptome data. We observed the expression levels of *TaJAZs* at different stages of wheat spike development. Most *TaJAZ* homologs were consistently expressed over the entire spike development period. Expression patterns of *Ae. tauschii AeJAZ2*, *7*, *4*, and *10* were similar to their ortholog groups in common wheat, i.e., genes in Group 4 (*TaJAZ2A*, *3B*, and *3D*), Group1 (*TaJAZ9A*, *8B*, and *19U*), Group 9 (*TaJAZ6A*, *5B*, and *5D*), and Group11 (*TaJAZ11A*, *11B*, and *10D*) that were lowly expressed or not detectable during spike development (Figure 4 and Figure 6). In contrast, six *AeJAZ* genes (*AeJAZ1*, *3*, *5*, *6*, *8*, and *9*) were highly expressed in *Ae. tauschii* leaves. The homologs in wheat, i.e., those in Group 6 (*TaJAZ1A*, *1B*, and *1D*), Group 3 (*TaJAZ3A*, *4B*, and *4D*), Group 5 (*TaJAZ5A*, *6B*, and *6D*), Group 8 (*TaJAZ4A*, *7B*, and *7D*), Group 10 (*TaJAZ7A*, *9B*, and *8D*), and Group 7 (*TaJAZ8A*, *10B*, and *9D*), also showed higher expression levels in wheat spikes. Such expression patterns indicated functional conservation of the *JAZ* genes during the evolution from ancestral species to hexaploid wheat. Interestingly, a number of *TaJAZ* genes with maximum expression levels at the crucial spike formation stage, Waddington stage 3 or the W3 stage [22], such as *TaJAZ1-A*, *-B*, *-D*, *TaJAZ3A*, and *TaJAZ4D*, maintained relatively high expression levels, but only one of them, *TaJAZ1-D*, was located within spike QTL intervals (Figure 6). 

Genes involved in tandem duplication, such as those on chromosomes 4 and 7, exhibited relatively lower expression patterns in wheat spikes. A similar expression pattern was found for *Ae. tauschii* tandem duplication *JAZ* genes. However, 20 of these tandem duplication genes were located within QTLs associated with spike phenotypes. Despite their relatively low expression levels, the cumulative expression abundance of tandem repeat genes, as detected by a pair of common primers to amplify all three homoeologs (Figure 6), was high, which may cause an effect on spike development.

### 2.6. JAZ Genes Were Preferentially Selected during Wheat Breeding for Yield

To explore the potential role of JAZ genes in wheat breeding, we then identified selection valleys during improvement of landraces to cultivars, using Fst, pi, and XPCLR values [23,24]. Among the top 5% of ranked genes that were considered to be selected in the breeding process, 12 TaJAZ genes were identified (Table 1). Among them, five were located within previously identified QTLs related to spike development (Supplementary Table S3). Ten of these TaJAZ genes were in the A subgenome, while only two were in the B subgenome (Table 1), suggesting asymmetric selection of these genes during wheat breeding. Interestingly, all tandemly duplicated genes on the A subgenome chromosomes 4 and 7 were located within the breeding selection intervals. Among 12 genes located within selection intervals, 9 were tandemly duplicated genes. The substantial proportion of tandem duplication genes may underscore the importance of these genetic loci in wheat improvement.

**Figure 7:**
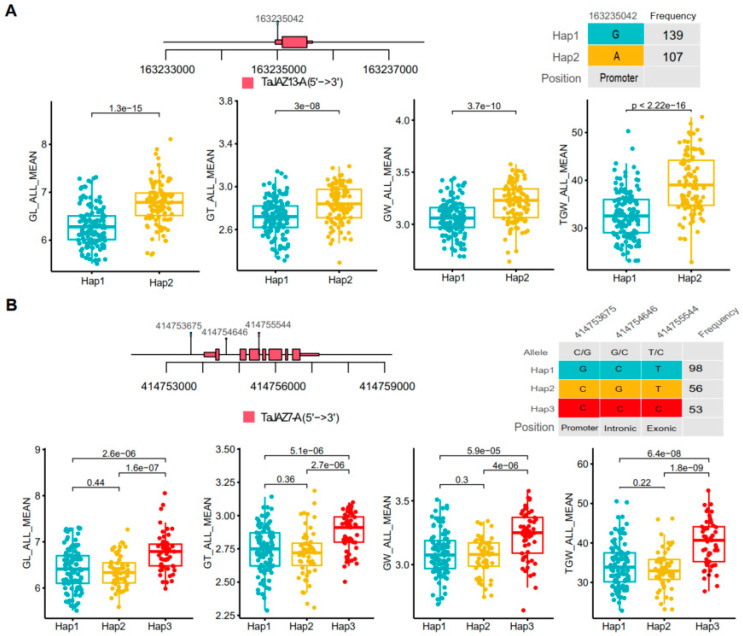
Haplotype analysis of *TaJAZ13A* and *TaJAZ7A* for agriculture traits. (**A**) Among the two *TaJAZ13A *haplotypes, Hap2 is associated with agronomic traits. (**B**) Among the three *TaJAZ7A* haplotypes, Hap3 is associated with agronomic traits in wheat. For each gene, the location of the SNP on the gene (**top left**) and haplotypes (**top right**) are shown, together with the association with agronomic traits. GL: grain length, GW: grain width, GT: grain thickness, TGW: thousand-grain weight. *p*-values were computed by the Student’s *t*-test.

We further studied the traits that may be affected by selected *JAZ* genes in wheat cultivars. Haplotype analysis showed that tandem duplication *JAZ* genes tend to exhibit significant differences with respect to multiple yield-related agronomic traits (Figure 7). For instance, accessions represented by the two haplotypes distinguished by an SNP at the promoter region of *TaJAZ13-A *showed significant differences in four yield-related agronomic traits, namely, grain length, grain thickness, grain width, and thousand-grain weight (TGW), with Hap2 displaying a higher TGW (*p* < 0.01) and larger kernels (*p* < 0.01), while Hap1 showed a lower TGW and smaller kernels (Figure 7A). In another case, three haplotypes (Hap1, -2, and -3) were derived from three SNPs located in the promoter, intron, and exon regions of *TaJAZ7-A*, respectively. Association analysis between these haplotypes and grain traits showed significant differences among the three haplotypes in terms of grain size and weight. As shown in Figure 7B, Hap3 represented accessions with a higher TGW (*p* < 0.01) and larger kernels (*p* < 0.01), while Hap1 and Hap2 had lower TGWs and smaller kernels. These data indicated that *JAZ* genes, such as *TaJAZ7-A* and *TaJAZ13-A*, may have significant applications in wheat yield improvement.

## 3. Discussion

The integration of *Ae. tauschii* with tetraploid wheat provided not only additional capability for biotic and abiotic resistance, but also better-quality end products for hexaploid common wheat. In light of the important roles of JA and *JAZ* genes in many aspects of plant development and adaptation, study of *JAZ *genes should provide new insights into the functions and application of these genes in wheat breeding improvement.

Although studies on *JAZ* genes in wheat have been conducted, these works have been limited by insufficient genome completeness and continuation. The availability of a high-quality reference genome overcame the problems of inaccurate genomic structures that prevented relatively precise analysis of gene replication events that led to the amplification of the family. Low-quality genome sequences may also result in less accurate gene annotation and a lack of quality population genomic data. In this work, we integrated the high-quality reference genomes for wheat and *Ae. tauschii* with comprehensive transcriptome data for wheat yield-related traits [18,19,20,25] and further improved the gene composition of the *JAZ* gene families in the two species. Further utilization of the population genomic data [23,24] allowed systematic analysis of the evolution and selection patterns during wheat improvement.

The improved reference genomes also facilitated more detailed analysis of the genomic structure of the gene family. Patterns for gene family expansion were observed, and 28 out of 54 *TaJAZ* genes were found to have resulted mainly from tandem or proximal duplications. Monocots, including *Ae. tauschii*, contained more *JAZ* genes that were mostly generated by tandem duplication. Five new *JAZ *gene members were identified in common wheat, suggesting novel roles of these genes in both natural variation and breeding selection. More interestingly, these tandemly repeated *JAZ *members tended to be located in genomic intervals covered by known QTLs associated with agronomic traits like yield, indicating that these JA-associated genes were preferentially selected during breeding.

Transcriptome analysis of *Ae. tauschii *seedling leaves after JA treatment identified 12 *AeJAZ* genes that were significantly upregulated after JA treatment. To demonstrate the accuracy of the results, in comparison with previous transcriptome studies of *Ae. tauschii*, we found that the tandemly duplicated *AeJAZ* genes on chromosome 7 that cannot be induced by JA were hardly expressed in spikes (Table S4). Interestingly, *TaJAZ* orthologs of *AeJAZ *can be induced by JA, which also exhibited consistently high expression in wheat spikes, whereas others remained silent or were expressed at low levels. However, unlike in diploids, the tandemly duplicated *TaJAZ* genes located on chromosome 7 in wheat can be induced by JA (Table S5). JA-induced RNA-seq analysis identified a total of 48,556 genes that responded to JA treatment that significantly enhanced the annotation rate of the genome in *Ae. tauschii*., which may assist the further functional analysis of other genes in JA signaling pathways. Moreover, *JAZ* genes were frequently found within selection valleys or associated with agronomic traits, as shown by the favorable allelic variations in *TaJAZ7A* and *TaJAZ13A* that were significantly associated with yield in wheat. With more mechanistic study of wheat JA signaling pathways and the deepening of our understanding of the function of *JAZ* genes [26], more approaches can be explored to make use of these important genes for wheat yield improvement, which is essential for world food security.

## 4. Materials and Methods

### 4.1. Sequence Analysis of JAZ Genes

A two-step method was used to identify *JAZ* genes in *Ae. tauschii* and *T. aestivum*. Firstly, a BLAST search was conducted using all *A. thaliana*, *Brachypodium distachyon*, and rice JAZ protein sequences as queries against the *Ae. tauschii* and newest wheat reference genome sequences, respectively. The resulting protein sequences with an e-value < 10^−5^ were collected, and duplicates were removed. These JAZ protein sequences were then examined for the presence of TIFY and Jas domains using InterProScan and the Pfam database, respectively. The genomic and coding sequences of the identified *Ae. tauschii *and *T. aestivum JAZ* genes were obtained from Phytozome and analyzed for exon/intron organization using TBtools-II [27]. The physical and chemical properties of each AeJAZ protein were predicted using the ExPasy program [28], and subcellular localization was predicted through the LOCALIZER online program [29]. To create a genetic map, chromosomal locations of *AeJAZ* genes obtained from Phytozome were drawn using the chromoMap package in R 4.1 [30].

### 4.2. Conserved Motif Identification

The MEME program was used to annotate the structural motifs of all *Ae. tauschii* and *T. aestivum JAZ *proteins. The alignment of protein sequences was manually checked [31]. The online tool weblogo was utilized to generate sequence logos for the conserved regions found. 

### 4.3. Phylogenetic Analysis

To study the evolutionary relationship of AeJAZ proteins, a total of 106 JAZ proteins from seven different plants were analyzed, including monocot plants (*Oryza sativa*, *Sorghum bicolor*, *B. distachyum*, and *Z. mays*) and eudicots (*A. thaliana *and *Solanum lycopersicum*). Protein sequences were obtained from Ensemble plant and Phytozome 33. A phylogenetic tree was created using MEGA X with the maximum likelihood method with the Poisson correction model and 1000 bootstrap values [32].

### 4.4. Plant Materials, RNA Extraction, Library Construction, and Illumina Sequencing

The *Ae. tauschii* accession Y2282 was grown under the long-day condition (24 °C, 16 h light/8 h dark). Seedlings that were 14 days old were treated with 5 mM MeJA solution, and double-distilled H_2_O (ddH_2_O) was used as a mock treatment. Shoots and leaves were isolated after applying JA solution and ddH_2_O for 1 h and 6 h, respectively (Figure S1). Each biological replicate contained 6~7 individual plants. Tissues were harvested at 10 o’clock in the morning daily to synchronize circadian effects, and the samples were stored in liquid nitrogen immediately. Total RNA was extracted using Trizol reagent (Invitrogen, Waltham, MA, USA) and treated with TURBO DNase I (Ambion, Austin, TX, USA) for 30 min and purified using the RNeasy^®^ Plant Mini Kit (QIAGEN, Hilden, Germany). RNA sequence libraries were prepared using the TruSeq RNA sample Prep V2 kit according to the manufacturer’s instructions (Illumina, Inc., San Diego, CA, USA). The quality and size of cDNA libraries for sequencing were checked with the Agilent 2200 TapeStation system (Agilent Inc., Santa Clara, CA, USA). RNA libraries were sequenced using the HiSeqX 10 sequencer system (Illumina Inc., San Diego, CA, USA) with a 150-cycle paired-end sequencing protocol.

### 4.5. Transcriptome Data Analysis

Raw reads in FASTQ format were trimmed to obtained clean reads through trimmomatic [33] by filtering adapter-only reads, removing reads containing > 10% poly-N and low-quality reads with PHRED quality scores ≥ 20 [34]. High-quality clean data were calculated using FastQC. 

Paired-end reads were aligned to the *Ae. tauschii* genome (Aet_v4.0) by Hisat2 [35]. Aligned reads were summarized over gene models containing the annotated and unannotated transcripts using HTSeq-count with the following parameter setting: htseq-count -f bam -r name -s no -a 10 -n 10 -t exon -i gene_id -m union [36]. Read counts of genes were depth-adjusted using DESeq2 [37]. Differentially expressed genes (DEGs) were filtered with *p*-values less than 0.05 and absolute fold changes of more than two when performing pairwise comparisons between samples. 

The expression data for *JAZ* genes during spike development and spike-related QTL data were downloaded from the Wheat Spike Multi-Omics Database (WSMOD). The expression data included expression profiles at six stages of spike development: SAM (shoot apical meristem), W1.5, W2, W3, W3.25, and W4 [25].

### 4.6. Breeding Selection and Haplotype Comparative Analysis

Selection loci during breeding were identified by diversity comparison between landraces and cultivars as described in previous research [23]. Then, JAZ gene haplotypes were associated with yield traits. Genotypes were extracted from total data by utilizing vcftools and geneHapR to construct haplotypes for *JAZ* genes [38]. Subsequently, differential agronomic traits were associated with the two haplotypes.

## 5. Conclusions

This study further identified or updated the number of JAZ gene family members in Triticeae species and analyzed the expression of tandemly duplicated genes under hormone and salt stress treatment. Selection analysis within the wheat mini core population indicated that some *TaJAZ* genes are in the selection sweep and play an important role in wheat production improvement. These data provide information for wheat breeding and improvement.

## Figures and Tables

**Table 1:** TaJAZ genes located within a selection sweep between landraces and cultivars.

Names	ID	Window	Method	Value
TaJAZ4-A	TraesCS4A01G007800	Chr4A:4832295-4837294	Pi	0.341
TaJAZ5-A	TraesCS4A01G007900	Chr4A:4700001-4900000	Pi	0.341
TaJAZ6-A	TraesCS4A01G008000	Chr4A:4700001-4900000	Pi	0.341
TaJAZ7-A	TraesCS5A01G204900	Chr5A:414700001-415000000	Pi	0.330
TaJAZ10-B	TraesCS5B01G211000	Chr7A:381600001-382800000	Fst	5.06
TaJAZ12-A	TraesCS7A01G201100	Chr7A:162300001-164900000	Fst	10.12
TaJAZ13-A	TraesCS7A01G201200	Chr7A:162300001-164900000	Fst	10.12
TaJAZ14-A	TraesCS7A01G201300	Chr7A:162300001-164900000	Fst	10.12
TaJAZ15-A	TraesCS7A01G201400	Chr7A:162300001-164900000	Fst	10.12
TaJAZ16-A	TraesCS7A01G201500	Chr7A:162300001-164900000	Fst	10.12
TaJAZ17-A	TraesCS7A01G201600	Chr7A:162300001-164900000	Fst	10.12
TaJAZ12-B	TraesCS7B01G107700	Chr7B:124127813-124132812	XPCLR	6.55

## Data Availability

The transcriptome sequencing data have been submitted to GSA under the project number PRJCA023511.

## Supplementary Materials

The following supporting information can be downloaded at: https://www.mdpi.com/article/10.3390/plants13091259/s1, Figure S1: Characterization of *JAZ* genes in common wheat. Figure S2: Genome-wide identity of JA responsive genes. (A) Shoots of seedlings were isolated after 1-h and 6-h JA and ddH_2_O treatments. (B) Transcriptome sample correlation analysis. (C) Venn diagram shows differentially expressed genes by pairwise comparison. Figure S3: Strand-specific transcriptome increased the number of identified genes. (A) Over 98% of the sequences in the transcriptome exhibited strand specificity. (B) Identification of 43,580 known genes and 4976 unknown genes from JA-treated transcriptomes. Table S1: List of 17 *JAZ* genes in *Aegilops tauschii*. Table S2: List of 54 *JAZ* genes in common wheat. Table S3: *AeJAZ* gene expression patterns under methyl jasmonate treatment. Table S4: The expression levels of 17 *AeJAZ* genes in spikes, seedlings, and seeds of *Aegilops tauschii*. Table S5: *TaJAZ* genes induced by JA treatment.
