# Supplementary figures and images for: Comprehensive Comparative Analysis of the JAZ Gene Family in Common Wheat (Triticum aestivum) and Its D-Subgenome Donor Aegilops tauschii
Source: Plants (Basel). 2024 Apr 30;13(9):1259. doi: 10.3390/plants13091259 (PMC11085061; doi:10.3390/plants13091259)

**Figure S1**

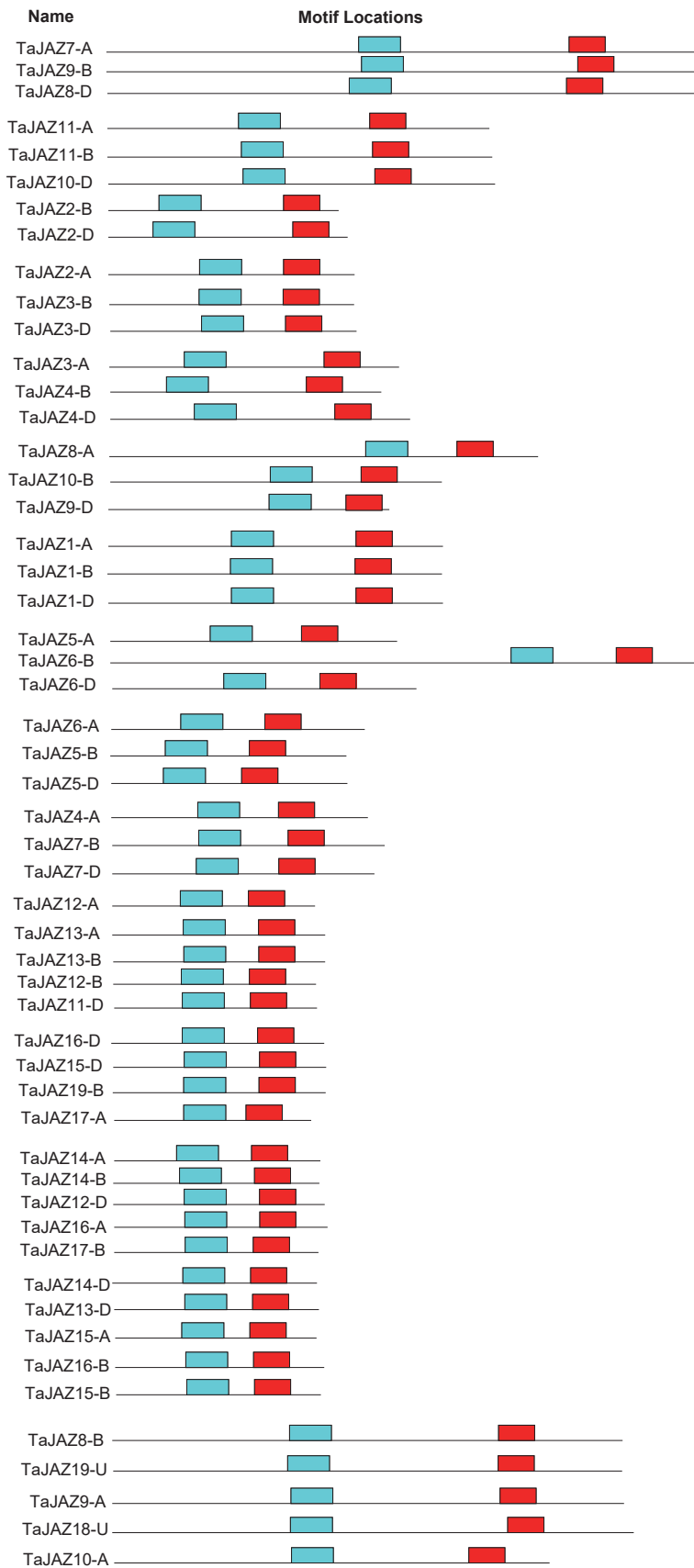

Supplement: Supplementary file 1 [file plants-13-01259-s001.zip › figureS1.pdf]

Figure S2

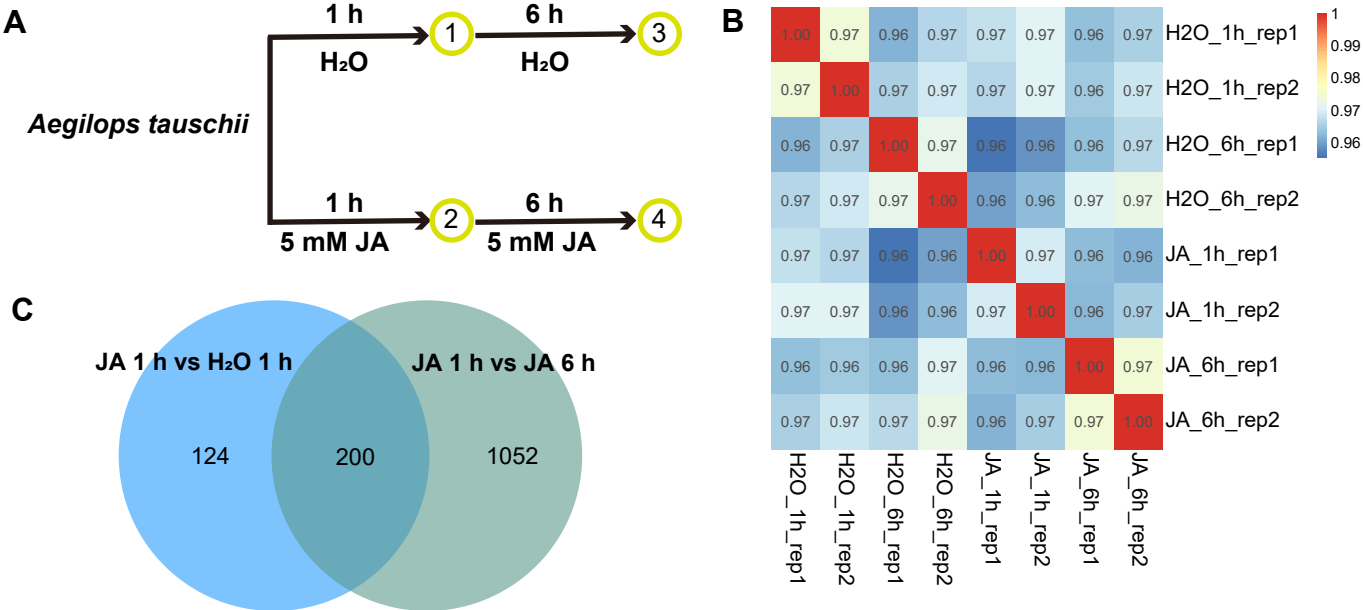

Supplement: Supplementary file 1 [file plants-13-01259-s001.zip › figureS2.pdf]

Figure S3

A

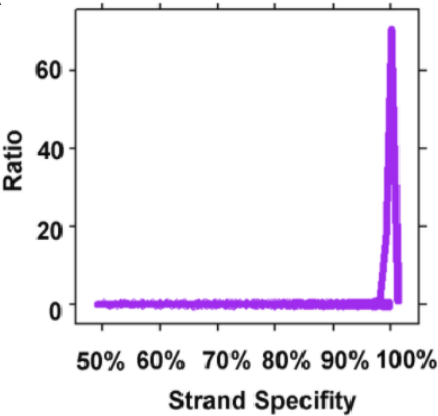

B

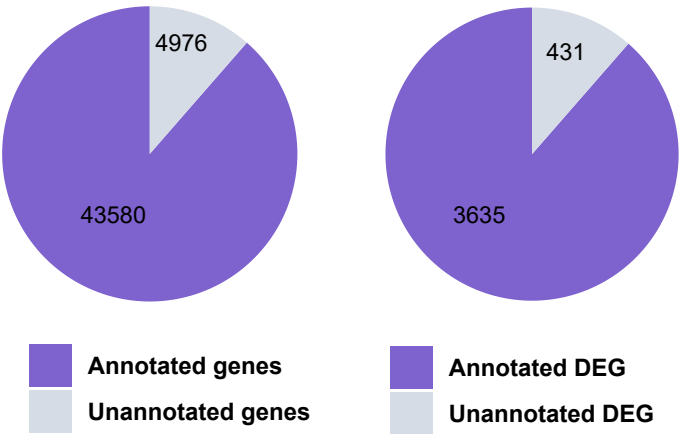

Supplement: Supplementary file 1 [file plants-13-01259-s001.zip › figureS3.pdf]
